# Identification and Analysis of *BCAS4*/*hsa-miR-185-5p*/*SHISA7* Competing Endogenous RNA Axis in Late-Onset Alzheimer’s Disease Using Bioinformatic and Experimental Approaches

**DOI:** 10.3389/fnagi.2022.812169

**Published:** 2022-02-21

**Authors:** Hani Sabaie, Mahnaz Talebi, Jalal Gharesouarn, Mohammad Reza Asadi, Abbas Jalaiei, Shahram Arsang-Jang, Bashdar Mahmud Hussen, Mohammad Taheri, Reza Jalili Khoshnoud, Maryam Rezazadeh

**Affiliations:** ^1^Clinical Research Development Unit of Tabriz Valiasr Hospital, Tabriz University of Medical Sciences, Tabriz, Iran; ^2^Department of Medical Genetics, Faculty of Medicine, Tabriz University of Medical Sciences, Tabriz, Iran; ^3^Neurosciences Research Center (NSRC), Tabriz University of Medical Sciences, Tabriz, Iran; ^4^Cancer Gene Therapy Research Center, Zanjan University of Medical Sciences, Zanjan, Iran; ^5^Department of Pharmacognosy, College of Pharmacy, Hawler Medical University, Erbil, Iraq; ^6^Center of Research and Strategic Studies, Lebanese French University, Erbil, Iraq; ^7^Institute of Human Genetics, Jena University Hospital, Jena, Germany; ^8^Skull Base Research Center, Loghman Hakim Hospital, Shahid Beheshti University of Medical Sciences, Tehran, Iran; ^9^Functional Neurosurgery Research Center, Shahid Beheshti University of Medical Sciences, Tehran, Iran

**Keywords:** Alzheimer’s disease, *BCAS4*, competing endogenous RNA, miR-185, *SHISA7*

## Abstract

Alzheimer’s disease (AD) is a heterogeneous degenerative brain disorder with a rising prevalence worldwide. *SHISA7* (*CKAMP59*) has emerged as one of the most intriguing new members of the SHISA family, in that, unlike other CKAMP counterparts, it exhibits a direct function in inhibitory synaptic GABAAR regulation. We used bioinformatics and experimental methods in this research to explore competing endogenous RNA (ceRNA) regulation of *BCAS4* and *SHISA7* in tau pathogenesis and their capacity as peripheral biomarkers linked to an abnormal inflammatory response in AD. The Gene Expression Omnibus database included two microarray datasets, including information on mRNAs (GSE106241) and miRNAs (GSE157239) from individuals with AD with different degrees of AD-associated neurofibrillary pathology in the temporal cortex (TC) tissue specimens and corresponding controls were downloaded from the Gene Expression Omnibus database. The limma package in the R software was used to identify differently expressed mRNAs (DEmRNAs) and miRNAs (DEmiRNAs) associated with AD-related neurofibrillary pathology. Additionally, we used the quantitative polymerase chain reaction technique to examine the expression of the *BCAS4*/*hsa-miR-185-5p*/*SHISA7* ceRNA axis in the peripheral blood (PB) of fifty AD patients and fifty control subjects. *BCAS4* was shown to act as a ceRNA to control the *SHISA7* expression throughout AD-associated neurofibrillary pathology in TC tissue specimens by sponging *hsa-miR-185-5p*, based on our bioinformatics study. Furthermore, in PB specimens from individuals suffering from AD and normal controls, we found no substantial differences in *BCAS4* expression patterns. *SHISA7* expression in AD patients’ PB was found to be reduced, as was the case in the TC. On the other hand, we discovered reduced amounts of *hsa-miR-185-5p* in AD patients’ PB samples compared to control subjects, unlike in TC tissue, where it had been demonstrated to be overexpressed. *BCAS4* and *SHISA7* expression levels showed a strong positive correlation, suggesting the presence of an interconnected network, most likely as a result of ceRNA regulation among PB specimens. The present study is the first evidence to highlight the expression of the *BCAS4*/*miR-185-5p*/*SHISA7* ceRNA axis in the brain and PB of AD patients, and offers a new viewpoint on molecular processes underlying AD pathogenic mechanisms.

## Introduction

Alzheimer’s disease (AD) is known as a type of dementia and a progressive neurodegenerative disorder (NDD), causing memory, thinking, and behavioral problems (Kang et al., 2020). According to the Alzheimer’s Association, AD accounts for 60–80 percent of dementia people. Nowadays, 50 million individuals worldwide suffer from AD and other types of dementia. After the age of 65, every 5 years, the incidence of AD doubles (Fan et al., 2019). Symptoms usually appear gradually and worsen with time. Regarding genetic aspects, AD is a heterogeneous polygenic disorder. The illness has been divided into two types based on the age of onset: early-onset AD (EOAD) and late-onset AD (LOAD). The most prevalent type of dementia is LOAD, commonly known as sporadic AD (SAD). AD is a complicated disease caused by susceptible genes and also environmental variables (Rezazadeh et al., 2019). Epigenetic alterations such as non-coding RNA regulation, DNA methylation, and histone modification may all impact tau phosphorylation regulation directly or indirectly, hence contributing to the development and progression of AD (Yu et al., 2019; Noroozi et al., 2021). On the other hand, genes have an important influence on AD. LOAD has a heritability of 58–79 percent, whereas EOAD has over 90 percent. The genetic association studies have helped us comprehend the etiology of AD. There are now around 50 loci linked to AD. These data strongly imply that AD is a complicated illness (Sims et al., 2020). The buildup of β-amyloid peptide (Aβ) within the brain and hyperphosphorylated and cleaved microtubule-associated protein tau are two core pathologies of AD. It is documented that neurofibrillary tangles (NFTs) and senile plaques are formed due to metabolic malfunction of Aβ precursor protein (APP) and aberrant phosphorylation of the protein tau (Kang et al., 2020) or maybe their interaction with each other (Busche and Hyman, 2020). Genetic, biochemical, and behavioral studies show that the pathologic formation of the neurotoxic Aβ peptide following serial APP proteolysis is a key stage in AD pathogenesis. Furthermore, APP is quickly and intricately processed by sequential secretases, including β-site APP-cleaving enzyme 1 (BACE1), γ-secretase, and the ADAM family as α-secretases. In terms of tau proteolysis, this mechanism is critical in tau aggregation and neurodegeneration. The Microtubule-Associated Protein Tau (*MAPT*) gene encodes tau, a microtubule-associated protein primarily generated in neurons. Intracellular tau is occasionally hyperphosphorylated, forming dangerous oligomers and observable aggregates as NFTs (Kang et al., 2020). During the last decade, a third fundamental characteristic of AD has arisen, which may give insight into the pathogenesis of AD and a connection between the two types of main pathologies (Kinney et al., 2018). The pathogenesis of AD affects a variety of inflammatory molecules, with aberrant amounts in several brain areas (Akiyama et al., 2000; Wilson et al., 2002). Aβ and APP stimulate the production of cytokines and chemokines from neurons, microglia, and astrocytes; chemokines and cytokines also increase the synthesis and accumulation of Aβ, therefore activating the vicious cycle (Solfrizzi et al., 2006). Moreover, neuroinflammation, defined by microglial activation prior to tau tangle development, might be an early occurrence and play a critical role in the pathology of tau. In P301S transgenic mice, immunosuppression improved tau pathology (Metcalfe and Figueiredo-Pereira, 2010). Furthermore, the peripheral level of inflammation-associated cytokines varies throughout the pathogenesis of AD and is substantially associated with disease development (Motta et al., 2007; Holmes et al., 2009; Leung et al., 2013). As a result, to decipher the mechanism of the pathogenesis of AD, the crosstalk between aberrant phenomena in the central and the peripheral immune system should be understood (Dionisio-Santos et al., 2019; Park et al., 2020). Irrespective of the pathological features, synaptic dysfunction is largely regarded as a causative phenomenon in AD. There are two primary synapses (glutamatergic and GABAergic) in the central nervous system (CNS), which produce excitatory and inhibitory responses, respectively. A large body of evidence suggests that the glutamatergic system is compromised throughout disease progression. Nonetheless, new data suggest that the GABAergic pathway experiences pathogenic changes and contributes to AD development (Li et al., 2016).

*SHISA7* (*CKAMP59*) has emerged as an intriguing SHISA family because, unlike other CKAMPs, Shisa7 directly affects the regulation of GABAARs at inhibitory synapses (Castellano et al., 2021). It co-localizes in hippocampal neurons with GABAARs and gephyrin (Castellano et al., 2021), whereas other CKAMPs are found at glutamatergic synapses (von Engelhardt et al., 2010; Klaassen et al., 2016; Peter et al., 2020). Moreover, it controlled GABAAR trafficking and inhibitory transmission while having no effect on excitatory synaptic transmission. Interestingly, Shisa7 influences GABAAR kinetics as well as its pharmacological characteristics (Castellano et al., 2021). The activation of the GABAAR may promote tau phosphorylation by decreasing the interaction of protein phosphatase 2A (PP2A) with tau, increasing intracellular NFTs within neurons and contributing to AD development. Hyperphosphorylated tau, on the other hand, may increase GABAergic neurotransmission. In the neurological system, there may be a feedback loop between the activation of the GABAAR and phosphorylation of tau (Li et al., 2016). Furthermore, recent research demonstrates the dual involvement of the GABAA receptor in neuroinflammation and peripheral inflammation, implying a complex interplay between GABAergic systems and immunity systems (Malaguarnera et al., 2021). To the best of our knowledge, regulating the expression of *SHISA7* in AD development has not been investigated yet. Salmena et al. (2011) suggested a novel regulatory mechanism termed competing endogenous RNA (ceRNA). This hypothesis refers to the existence of a reversed RNA → microRNA function, in which RNAs actively control one another via direct competition for miRNA complementary sequences known as miRNA response elements (MREs) (Salmena et al., 2011; Tay et al., 2011). According to the ceRNA hypothesis, if two RNA transcripts control one another via a ceRNA-mediated mechanism, the expression of the two RNA transcripts will correlate negatively with the expression level of target miRNAs and positively with one another (Salmena et al., 2011). Several investigations in recent years have confirmed the ceRNA hypothesis. Disruptions to the ceRNA crosstalk equilibrium are well recognized to be involved in NDDs. In addition, ceRNAs were studied as biomarkers in NDDs (Moreno-García et al., 2020). Amongst NDDs, ceRNA interactions in AD have received a great deal of attention (Cai and Wan, 2018). A study on the human cell line indicated that *BCAS4*, as a *SHISA7* ceRNA, modulates the *SHISA7* expression level through a miRNA-dependent mechanism (Marques et al., 2012). Based on the as-mentioned theoretical concepts, the *BCAS4*/*SHISA7* ceRNA pair may be significantly involved in AD development.

This study aimed to examine the ceRNA regulation of *SHISA7* and *BCAS4* in tau pathogenesis and their usefulness as peripheral biomarkers associated with aberrant inflammation in the development of AD, according to bioinformatics and experimental approaches.

## Materials and Methods

### Bioinformatics Analysis Based on Brain Microarray Dataset

#### Gene Expression Profile Data Collection

In this research, a bioinformatics approach was employed to mine data from a microarray dataset of human temporal cortical (TC) tissue samples with different degrees of AD-related neurofibrillary pathology (GSE106241). We intended to identify expression changes of *SHISA7* and *BCAS4* in human TC tissue samples. The above gene expression profile was obtained from the NCBI Gene Expression Omnibus database (GEO^1^). A chip-based platform GPL24170 Agilent-044312 Human 8 × 60K Custom Exon array (Probe Name version) was used for the dataset. The GSE106241 included 60 human TC tissue samples split into seven groups according to Braak staging (Braak 0: *n* = 6, Braak I: *n* = 11, Braak II: *n* = 11, Braak III: *n* = 6, Braak IV: *n* = 7, Braak V: *n* = 12, Braak VI: *n* = 7) reflecting the severity of the disease (Marttinen et al., 2019). Braak staging is a type of neuropathological staging that distinguishes between early, middle, and advanced AD according to the development of NFTs within the medial temporal lobe memory circuit. Braak Stage 0 corresponds to the absence of NFTs, Stages I-II to entorhinal-perirhinal cortex NFTs, Stages III-IV to NFTs additionally in the hippocampus, and Stages V-VI to NFTs dispersed across the neocortical regions (Braak and Braak, 1991).

#### Data Preprocessing and Differentially Expressed Genes (DEGs) Identification

Using the normexp method, background correction was performed (Silver et al., 2009) with an offset of 15, and between-array normalization was performed utilizing the quantile algorithm using normalizeBetweenArrays() function from linear models for microarray data (limma) R package (Ritchie et al., 2015). Only spots with signal minus background flagged as “positive and significant” (field name gIsPosAndSignif) and not flagged as ControlType or IsManualFlag were utilized. The AgiMicroRna Bioconductor package (version 2.40.0) was used to evaluate the quality. The principal component analysis (PCA) was used for a dimensional reduction analysis (Yeung and Ruzzo, 2001) to find similarities between each sample group by the ggplot2 package in R software version 4.0.3. Differentially expressed gene analysis (DEGA) was performed comparing AD samples with normal samples using the linear models for microarray data (limma) R package (Ritchie et al., 2015) in Bioconductor^2^ (Huber et al., 2015). Student’s *t*-test was used to identify statistically significant genes. Also, the cut-off for aberrantly expressed mRNAs was established as follows: (Kang et al., 2020) a false discovery rate (adjusted *P*-value) < 0.01, and (Fan et al., 2019) | log2 fold change (log2FC)| > 0.3. The volcano plot for DEGs and heat map for *BCAS4* and *SHISA7* genes were created using the Enhanced Volcano (version 1.8.0) and Pheatmap (version 1.0.12) R packages.

#### Identification of miRNAs Associated With Alzheimer’s Disease-Related Neurofibrillary Pathology

To discover the differentially expressed miRNAs (DEmiRNAs) linked to AD-related neurofibrillary pathology, we employed a bioinformatics method identical to the one described above. GSE157239 miRNA profile data were acquired from the NCBI GEO. The platform GPL21572 (miRNA-4) Affymetrix Multispecies miRNA-4 Array (ProbeSet ID version) was applied for the dataset. The GSE157239 comprised eight TC samples from AD patients (Braak stage III or above) and eight from control subjects. The Robust Multichip Average (RMA), an effective tool in the affy Bioconductor package, was used for background correction and quantile normalization of the entire raw data files (Irizarry et al., 2003). An interquartile range filter (IQR across the samples on the log base two scale greater than median IQR) was used to lower the number of analyzed genes, which was followed by an intensity filter (a minimum of >100 expression signals in a minimum of 25% of the arrays) to remove insignificant probe sets that are not expressed or changing (von Heydebreck et al., 2005). The quality was assessed by the AgiMicroRna Bioconductor package. The PCA was used for a dimensional reduction analysis (Yeung and Ruzzo, 2001) by the ggplot2 package in R software. The DEGA was conducted using the limma R package (Ritchie et al., 2015) in Bioconductor (Huber et al., 2015) on normal and AD samples. To transform the miRNA names to miRbase v22, the miRNAmeConverter Bioconductor package (Haunsberger et al., 2017) was utilized. Student’s *t*-test was used to identify statistically significant miRNAs. Also, the cut-off for aberrantly expressed miRNAs were set as follows: (Kang et al., 2020) a false discovery rate (adjusted *P*-value) < 0.01, and (Fan et al., 2019) | log2 fold change (log2FC)| > 0.3. The DEmiRNA heat map was created utilizing R’s Pheatmap package.

#### Prediction of miRNA-mRNA Interactions

We used miRWalk (version 3) to identify interactions among miRNAs linked to AD-related neurofibrillary pathology with *BCAS4*/*SHISA7* (Sticht et al., 2018). Binding sites 3′UTR and score ≥ 0.95 were considered as criteria for the miRWalk query.

#### Correlation Analysis Between *BCAS4* and *SHISA7*, and Competing Endogenous RNA Axes Construction

The Pearson correlation analysis was performed to determine if there were any positive correlations between *BCAS4* and *SHISA7* in the ceRNA regulatory axes. The Hmisc and psych packages were used to calculate the correlations and visualization. The ceRNA regulatory axes were built according to the co-expression relation and miRNA-mRNA interactions.

### Identification and Differential Analysis of *BCAS4*/*SHISA7* Competing Endogenous RNA Axis as an Inflammatory Biomarker in Peripheral Blood

#### Participants and Peripheral Blood Samples

This case-control research included 100 individuals, 50 AD patients as well as 50 healthy controls who were gender and age-matched. The present investigation was approved by Tabriz University of Medical Sciences’ clinical research ethics committee (Ethical code: IR.TBZMED.REC.1398.1264). The participants were recruited from the Department of Neurology of Tabriz University of Medical Sciences’ Imam Reza Hospital. A neurology specialist identified the individuals using the Diagnostic and Statistical Manual of the American Psychiatric Association (DSM-V) criteria (Association, 2013). The criteria for inclusion were 65 years of age and older and no other psychiatric/neurologic diagnoses other than AD. The control group was chosen from aged 65 years and above individuals without AD in the same department. Diabetes, active or chronic infectious diseases, thyroid disorders, cancer, renal and liver failure, inflammatory diseases or receiving anti-inflammatory drugs, metabolic disease, severe ischemic heart disease, alcohol abuse, cerebrovascular accident, and having received corticosteroids in the past 8 weeks of evaluation were all exclusion criteria. Mini-mental state examination (MMSE) was used to assess cognitive ability in both groups. Written informed consent was obtained from all participants or their primary caregivers before enrolling in the research. Finally, 5-ml of peripheral blood was taken from each participant in EDTA-treated tubes.

#### Expression Assays

Total RNA was isolated from whole blood utilizing the Hybrid-R™ Blood RNA purification kit according to the manufacturer’s instructions (GeneALL, Seoul, South Korea) and treated with DNase I to remove DNA contamination. NanoDrop was employed to assess the quantity and quality of extracted RNA (Thermo Scientific, Wilmington, DE, United States). The synthesis of cDNA was done by the cDNA synthesis Kit (GeneALL) according to the manufacturer’s guidelines. The cDNA was stored at −20°C for further investigation. The primer sequences used in reverse transcription, as well as qPCR reactions, are listed in Table 1. *U6* and Ubiquitin C (*UBC*) were employed as internal controls to normalize miRNA and mRNA levels, respectively. The Step OnePlus™ Real-Time PCR and the RealQ Plus2x Master Mix (Ampliqon, Odense, Denmark) were used for the qPCR. All qPCR reactions were performed in duplicate.

**TABLE 1:** Sequences of primers used in reverse transcription (RT) and qPCR reactions.

Gene name	Gene reference ID	Primer sequences
*hsa-miR-185-5p*	–	RT primer: GTCGTATCCAGTGCAGGGTCCGAGGTATTCGCACTGGATACGACTCAGGAA Forward primer: AATCGGCGTGGAGAGAAAGGC Reverse primer: GTCGTATCCAGTGCAGGGTCC
*U6*	–	RT primer: GTCGTATCCAGTGCAGGGTCCGAGGTATTCGCACTGGATACGACAAAAATAT Forward primer: GCTTCGGCAGCACATATACTAAAAT Reverse primer: CGCTTCACGAATTTGCGTGTCAT
*BCAS4*	NM_198799.4 XM_017027932.1 XM_011528887.2 XM_011528886.2 NM_017843.4 NM_001010974.2	Forward primer: ATGCTCCTCAGGCTGGAAGAGT Reverse primer: CCACGCATTTCTGTCAGTTTGGC
*SHISA7*	NM_001145176.2	Forward primer: TGAAGACCCCCAACCTCGACTG Reverse primer: TCCTTCTCGGCCAGCCTCTTG
*UBC*	NM_021009.7	Forward primer: CAGCCGGGATTTGGGTCG Reverse primer: CACGAAGATCTGCATTGTCAAGT

#### Statistical Analysis for qPCR

The data analysis was performed using the R v.4 software packages brms, stan, pROC, and GGally. The Bayesian regression model was employed to investigate the relative expressions of *BCAS4*, *SHISA7*, and *hsa-miR-185-5p* in AD patients and healthy controls, as well as subgroups. Age and gender impacts were adjusted. The adjusted *P*-values of less than 0.05 were considered significant. The expression of the abovementioned genes was also studied across age groups and between males and females. The Spearman correlation coefficients were utilized to evaluate the connections between the variables in the research. The genes’ diagnostic power was determined using a receiver operating characteristic (ROC) curve analysis.

## Results

### Brain Microarray Dataset Reanalysis

#### Differentially Expressed Genes Identification

Background correction, batch modification, gene filtering, and normalization were all performed prior to DEGA. The AgiMicroRna Bioconductor package was utilized to monitor the quality. The gene expression dataset’s box plots were used to evaluate the distribution of data after normalization (Supplementary File 1). Distinct arrays in the box plots had similar expression level medians, suggesting that the correction was done properly. A PCA plot was also used to illustrate the spatial dispersion of samples (Supplementary File 1). PCA displays the specifics of the investigated data’s structure and aids in the discovery of similarities across samples.

**FIGURE 1:**
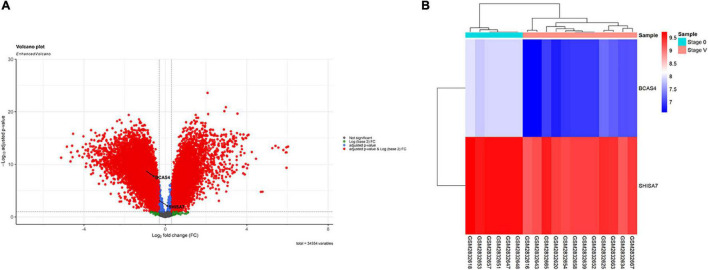
Differentially expressed mRNAs between brain samples of Alzheimer’s disease (AD) patients in Braak stage V and control (CTL) samples. **(A)** Volcano plot for the DEGs. The DEGs were screened based on a | (log2FC) | > 0.3 and an adjusted *P*-value < 0.01. **(B)** Heatmap for BCAS4 and SHISA7 genes. High expressed genes are shown in red, while those expressed at low levels are blue.

According to the results, *BCAS4* was significantly downregulated in Braak stages I-VI, while *SHISA7* was significantly downregulated only in Braak stage V. Thus, Braak stage V was the only one in which both the *BCAS4* (log2FC = −0.95, adj.P.Val = 1.71E-09) and the *SHISA7* (log2FC = −0.32, adj.P.Val = 0.000685865) genes were dysregulated. Supplementary File 2 summarizes the information of the genes addressed in each step. A total of 18,590 DEGs were found in patients in Braak stage V using the criteria of adjusted *P*-value < 0.01, and (Fan et al., 2019) | log2 fold change (log2FC)| > 0.3. Figure 1 depicts a volcano plot of DEGs as well as a hierarchical clustering heatmap of *BCAS4* and *SHISA7* genes in Braak stage V.

#### Identification of miRNAs Associated With Alzheimer’s Disease-Related Neurofibrillary Pathology

**FIGURE 2:**
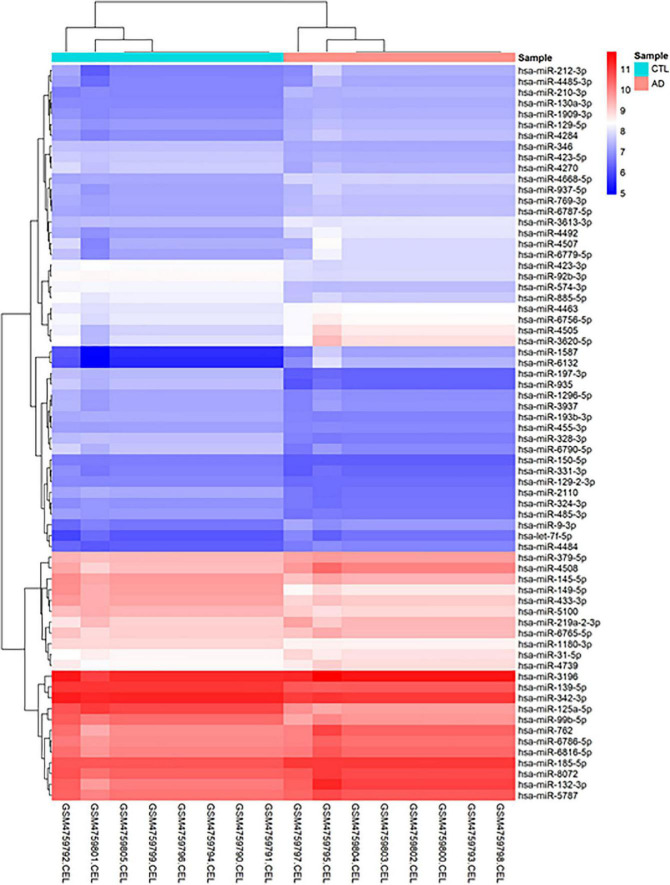
Hierarchical clustering heatmap differentially expressed human miRNAs between brain samples of Alzheimer’s disease (AD) and control (CTL) samples. High expressed genes are shown in red, while those expressed at low levels are blue.

Before conducting DEGA, gene filtering, normalization, batch adjustment, and background correction were performed. The AgiMicroRna Bioconductor program was utilized to monitor the quality. Box plots for gene expression profiles were shown after normalization to evaluate the distribution of data (Supplementary File 1). Distinct arrays in the box plots had similar expression level medians, suggesting a proper correction. In addition, a PCA plot was utilized to depict the distribution pattern of samples (Supplementary File 1). PCA displays the specifics of the investigated data structure. It also aids in determining the similarity of samples. A total of 68 human DEmiRNAs (38 up-regulated and 30 down-regulated) were discovered in GSE157239 comparing AD and control TC samples using the criteria [adjusted *P*-value < 0.01, and (Fan et al., 2019) | log2 fold change (log2FC)| > 0.3]. Figure 2 depicts a hierarchical clustering heatmap of DEmiRNAs. The details of DEmiRNAs are summarized in Supplementary File 2.

#### Prediction of miRNA-mRNA Interactions

We employed miRWalk database to find interactions between miRNAs linked to AD-related neurofibrillary pathology and *BCAS4*/*SHISA7* and later showed that three (*hsa-miR-185-5p*, *hsa-miR-423-5p*, *hsa-miR-5787*) and 14 miRNAs (*hsa-miR-145-5p*, *hsa-miR-150-5p*, *hsa-miR-185-5p*, *hsa-miR-3620-5p*, *hsa-miR-4270*, *hsa-miR-4463*, *hsa-miR-4507*, *hsa-miR-4508*, *hsa-miR-4739*, *hsa-miR-485-3p*, *hsa-miR-5100*, *hsa-miR-762*, *hsa-miR-769-3p*, *hsa-miR-937-5p*) obtained from GSE157239 dataset reanalysis may target *BCAS4* and *SHISA7*, respectively. Of those miRNAs, *hsa-miR-185-5p* targeted both genes *BCAS4* and *SHISA7*. The *hsa-miR-185-5p* expression levels in TC samples of AD were statistically higher than those in controls (log2FC = 0.37, adj.P.Val = 7.89E-24).

#### Correlation Analysis Between *BCAS4* and *SHISA7*, and Competing Endogenous RNA Axis Construction

**FIGURE 3:**
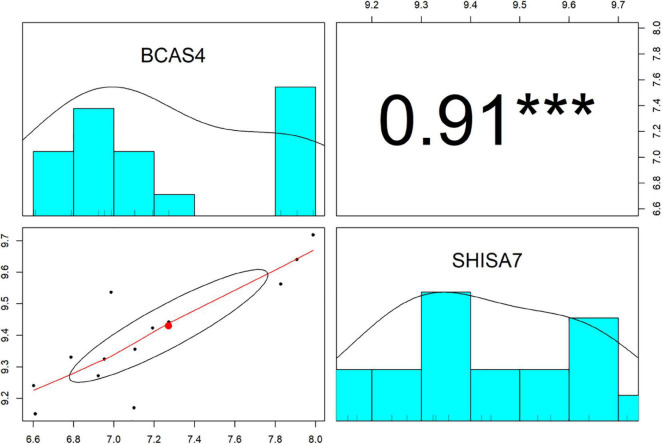
The distribution of each variable is shown on the diagonal. The lower portion of the diagonal shows bivariate scatter plots with a fitted line. On the upper part of the diagonal, the correlation coefficients plus the significance level as stars are displayed. *** is significant correlation at *P*-value < 0.001.

The Pearson correlation analysis was conducted between *BCAS4* and *SHISA7* to validate the ceRNA axes theory, which states that ceRNAs are positively regulated by each other via interactions with miRNAs. A significant positive correlation was found between the levels of expression of the evaluated genes (*r* = 0.91, *P* < 0.001) (Figure 3). Based on the co-expression and miRNA-mRNA interactions, *BCAS4* was shown to act as a ceRNA to control the *SHISA7* expression via sponging *hsa-miR-185-5p*.

### Identification and Differential Analysis of *BCAS4*/*hsa-miR-185-5p*/*SHISA7* Competing Endogenous RNA Axis as an Inflammatory Biomarker in Peripheral Blood

#### General Demographic Data

Following consideration of the inclusion and exclusion criteria, 50 AD patients (male/female%: 31.4/68.6) with age [mean ± standard deviation (SD)] of 76.36 ± 6.26 and 50 healthy controls (male/female%: 30.6/69.4) with age (mean ± SD) of 74.3 ± 6.22 were included in the study. The MMSE scores (mean ± SD) of the patient and control groups were 14.2 ± 5.94 and 27.5 ± 0.76, respectively.

#### Expression Assays

**FIGURE 4:**
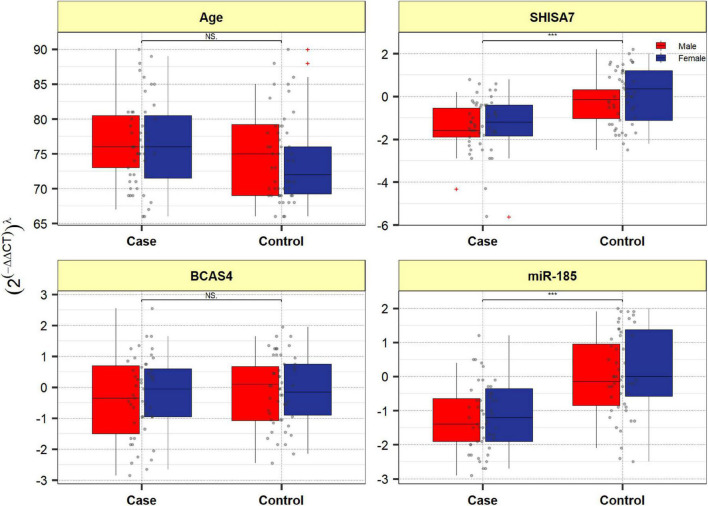
Expression of BCAS4, SHISA7, and hsa-miR-185-5p in cases and controls’ peripheral blood samples. Values are depicted as gray dots. Means of expression levels and interquartile range are displayed.

Figure 4 depicts *BCAS4*, *SHISA7*, and *hsa-miR-185-5p* genes’ relative expression levels in patients with AD and controls. The *BCAS4* expression levels demonstrated no significant differences in PB samples among AD patients and healthy controls (adjusted *P*-value = 0.967), as well as subgroups. *SHISA7* expression was significantly lower in PB samples from AD patients compared to controls (posterior beta = −1.035, adjusted *P*-value < 0.003). Such decreased expression was found between male and female subgroups too (posterior beta = −0.927, adjusted *P*-value = 0.035 and posterior beta = −0.932, adjusted *P*-value = 0.022, respectively). The *hsa-miR-185-5p* expression was found to be significantly lower (posterior beta = −0.849, adjusted *P*-value < 0.029) when PB samples from AD patients were compared to controls. Male and female subgroups also had lower expression (posterior beta = −0.857, adjusted *P*-value = 0.01 and posterior beta = −0.857, adjusted *P*-value = 0.01, respectively). Tables 2–4 provide comprehensive data on relative expressions of *BCAS4*, *SHISA7*, and *hsa-miR-185-5p*, respectively.

**TABLE 2:** Relative levels of *BCAS4* in AD cases and controls according to the Bayesian quantile regression model.

	*BCAS4*	Posterior Beta of (2^(–ddct)^)^λ^	SE	Adjusted *P*-Value*	95% Crl for Beta
Total	Group, Case vs. control	−0.124	0.26	0.967	[−0.57, 0.42]
	Sex, Female vs. Male	−0.061	0.29	0.647	[−0.59, 0.51]
	Age (years)	−0.011	0.02	0.801	[−0.04, 0.02]
	Group * Sex	0.017	0.4	0.427	[−0.78, 0.76]
Male	Case vs. control	−0.138	0.17	0.495	[−0.48, 0.2]
	Age	−0.012	0.01	0.886	[−0.04, 0.02]
Female	Case vs. control	−0.132	0.17	>0.999	[−0.46, 0.22]
	Age	−0.012	0.01	0.686	[−0.04, 0.02]

*Estimated from frequentist methods; CrI: Credible interval, ^λ^: Power transformation value estimated from Box-cox or Yeo-Johnson method. AD, Alzheimer’s disease.

**TABLE 3:** Relative levels of *SHISA7* in AD cases and controls according to the Bayesian quantile regression model.

	*SHISA7*	Posterior Beta of (2^(–ddct)^)^λ^	SE	Adjusted *P*-Value*	95% Crl for Beta
Total	Group, Case vs. control	−1.035	0.23	0.003	[−1.48, −0.58]
	Sex, Female vs. Male	0.217	0.25	0.26	[−0.26, 0.68]
	Age (years)	0.015	0.02	0.175	[−0.02, 0.04]
	Group * Sex	0.2	0.37	0.868	[−0.51, 0.93]
Male	Case vs. control	−0.927	0.18	0.035	[−1.28, −0.55]
	Age	0.019	0.02	0.389	[−0.01, 0.05]
Female	Case vs. control	−0.932	0.18	0.022	[−1.29, −0.56]
	Age	0.019	0.02	0.601	[−0.01, 0.05]

*Estimated from frequentist methods; CrI: Credible interval, ^λ^: Power transformation value estimated from Box-cox or Yeo-Johnson method. AD, Alzheimer’s disease.

**TABLE 4:** Relative levels of *miR-185* in AD cases and controls according to the Bayesian quantile regression model.

	*miR-185*	Posterior Beta of (2^(–ddct)^)^λ^	SE	Adjusted *P*-Value*	95% Crl for Beta
Total	Group, Case vs. control	−0.849	0.21	0.029	[−1.28, −0.46]
	Sex, Female vs. Male	0.068	0.25	0.906	[−0.45, 0.55]
	Age (years)	0.016	0.01	0.195	[−0.01, 0.04]
	Group * Sex	−0.028	0.35	>0.999	[−0.69, 0.68]
Male	Case vs. control	−0.857	0.16	0.01	[−1.18, −0.56]
	Age	0.015	0.01	0.814	[−0.01, 0.04]
Female	Case vs. control	−0.857	0.15	0.01	[−1.17, −0.56]
	Age	0.014	0.01	0.288	[−0.01, 0.04]

*Estimated from frequentist methods; CrI: Credible interval, ^λ^: Power transformation value estimated from Box-cox or Yeo-Johnson method. AD, Alzheimer’s disease.

#### Correlation Analysis

**FIGURE 5:**
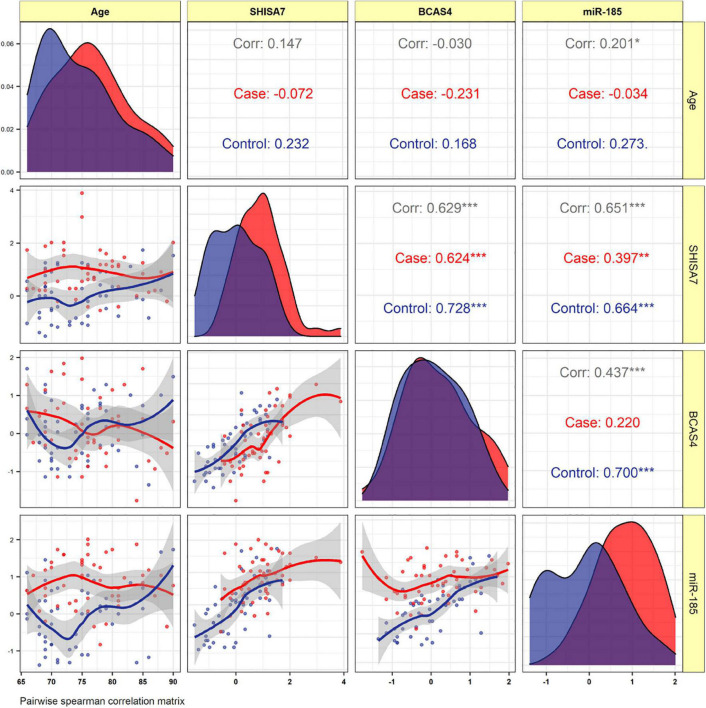
The distribution of variables is depicted on the diagonal. The correlation coefficients plus the significance level as stars are shown. *, **, and *** is significant correlation at *P* < 0.05, *P* < 0.01, and *P* < 0.001, respectively.

The *BCAS4*, *SHISA7*, and *hsa-miR-185-5p* expressions were not correlated to the patients’ age. In AD patients, expression of *BCAS4* were correlated positively with *SHISA7* (*r* = 0.624, *P* < 0.001). In the PB of patients, our correlation results between *hsa-miR-185-5p* and *SHISA7* showed a weak positive correlation (*r* = 0.397, *P* < 0.01) instead of a negative correlation. Furthermore, the expressed levels of *hsa-miR-185-5p* and *BCAS4* were not correlated significantly in AD cases (Figure 5).

#### Receiver Operating Characteristic Curve Analysis

**FIGURE 6:**
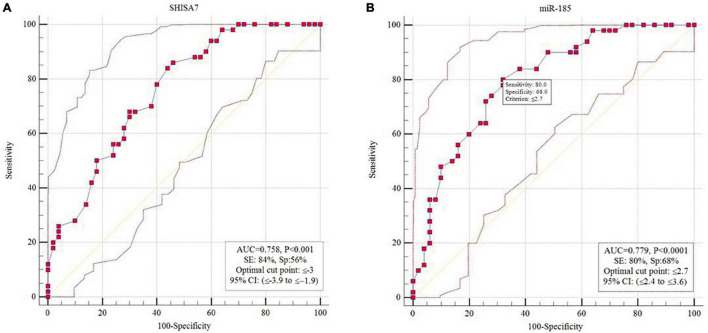
Receiver operating characteristic (ROC) curve analysis. **(A)** SHISA7 transcript levels displayed diagnostic power of 0.758. **(B)** Hsa-miR-185-5p transcript levels displayed diagnostic power of 0.779.

The diagnostic power of *SHISA7* and *hsa-miR-185-5p* were tested for their ability to distinguish AD patients from the controls. We obtained significant diagnostic powers of 0.758 and 0.779 from the transcript levels of *SHISA7* and *hsa-miR-185-5p*, respectively, by evaluating the area under the curve (AUC) (Figure 6).

## Discussion

According to growing research-based data, GABAergic neurotransmission faced serious pathological alterations in AD, and it might be a successful therapeutic candidate for this NDD (Li et al., 2016). Moreover, ceRNA regulation has biologically profound impacts in a variety of NDDs (e.g., Parkinson’s disease, AD, spinocerebellar ataxia type 7, amyotrophic lateral sclerosis, and multiple sclerosis), so it can explain the pathogenic processes and provides new options for therapy. Because of the multifactorial character of ceRNA interacting networks, they may be useful in the research of complicated diseases like AD. As a result, our efforts to comprehend various aspects of ceRNA regulation processes in AD pathology elucidate possible molecular targets, identify biomarkers based on ceRNA, and develop ceRNA-based therapeutic options (Cai and Wan, 2018; Moreno-García et al., 2020; Sabaie et al., 2021). In this study, we employed bioinformatics and experimental approaches to investigate the *BCAS4*/*SHISA7* verified ceRNA axis in AD development.

### *BCAS4*/*SHISA7* Competing Endogenous RNA Axis in Tau Pathology in Alzheimer’s Disease

Our bioinformatics analysis showed that *BCAS4* could serve as a ceRNA to regulate the expression of *SHISA7* in AD-related neurofibrillary pathology via sponging *hsa-miR-185-5p*. To our knowledge, this would be the first report of a possible role of the *BCAS4*/*hsa-miR-185-5p*/*SHISA7* axis in the tau pathology of AD.

Our *in silico* analysis showed that the expression levels of *BCAS4* were substantially lower in TC samples from AD patients and the control group. *BCAS4* is a new gene, which is cloned from breast cancer cells, encoding a cytoplasmic protein (211 aa) with no substantial homology to known proteins (Bärlund et al., 2002). A recent piece of research employing machine learning approaches and various microarray datasets revealed that *BCAS4* might be a ceRNA regulator for the development of intervertebral disc degeneration (Chang et al., 2020). To the best of our knowledge, this is the first study on *BCAS4* expression in TC samples of AD cases. In line with our results, a prior study on a human neuroblastoma cell line found that ceRNA regulation between *BCAS4* and *SHISA7* by *hsa-miR-185-5p* is conserved in humans (Marques et al., 2012).

Moreover, we discovered a decreased *SHISA7* expression level in TC samples from AD patients compared to controls. *Shisa7* controlled GABAAR trafficking and also inhibitory transmission while having no effect on excitatory synaptic transmission (Han et al., 2019). Interestingly, *Shisa7* influences the kinetics and pharmacological characteristics of the GABAAR. Although *Shisa7* lowered deactivation time constants for α1β2γ2 and α2β3γ2 receptors in heterologous cells, *Shisa7* KO increased decay time constants for GABAergic transmission in hippocampal neurons (Han et al., 2019). Eventually, *Shisa7* enhanced GABAAR potentiation induced by diazepam in heterologous cells, but *Shisa7* KO substantially decreased diazepam effects *in vivo* (Han et al., 2019). To the best of our knowledge, this is the first study on the expression level of *SHISA7* in AD patients. As previously stated, recent research using human neuroblastoma cell lines found that *hsa-miR-185-5p* regulates *BCAS4* and *SHISA7* in a ceRNA manner, which is consistent with our findings (Marques et al., 2012).

In addition to finding changed expression of *SHISA7* and *BCAS4* genes in tau pathogenesis, we discovered DEmiRNAs related to AD-associated neurofibrillary pathology. Among those miRNAs, just *hsa-miR-185-5p* targeted both *SHISA7* and *BCAS4* genes. Compared to controls, individuals with AD indicated higher *hsa-miR-185-5p* expression. In mice and humans, *MiR-185* mediates conserved crosstalk among *Pbcas4*, *BCAS4*, and protein-coding gene ceRNAs like *SHISA7* (Marques et al., 2012). A recent research study anticipated that the SNP rs5848 [C/T] created a 6 mer target site/MRE for *hsa-miR-185-5p* in the 3′UTR of granulin (proepithelin or *GRN*). They hypothesized that the development of this novel 6 mer MRE would lead to the observed *GRN* downregulation in LOAD (Roy and Mallick, 2017). The rs5848 GRN variant has been identified as a risk factor for AD in Taiwanese people (Lee et al., 2011), although the specific mechanism by which it affects the disease is unknown.

### *BCAS4*/*hsa-miR-185-5p*/*SHISA7* Competing Endogenous RNA Axis as an Inflammatory Biomarker in Peripheral Blood of Alzheimer’s Disease Patients

Due to the multifactorial nature of ceRNA interacting networks, they may be useful in investigations of complex NDDs like AD, particularly at levels of biomarkers (Moreno-García et al., 2020). As a result, we used qPCR to look into the expression level of the *BCAS4*/*hsa-miR-185-5p*/*SHISA7* ceRNA axis in our PB samples.

In PB samples, we found no significant differences in expression levels of *BCAS4* between AD patients and controls. Based on the literature, this is a study on the expression of *BCAS4* in PB samples from AD patients for the first time. Previous research has shown that the unique DNA methylation of *BCAS4* works as an epigenetic marker might be utilized to differentiate saliva from other types of body fluids. Moreover, it is utilized extensively in forensic investigations (Taki and Kibayashi, 2015; Silva et al., 2016).

*SHISA7* expression was reduced in the PB sample of patients with AD, just as it was in brain tissue. The current study is the first report considering the expression level of *SHISA7* in PB samples from AD patients. This concurrent reduction in the *SHISA7* expression level in the PB and brain might represent changes in AD patients’ brains in their periphery, making it a promising candidate for biomarker research (Ma et al., 2019).

Besides, in contrast to TC tissue, where *hsa-miR-185-5p* was found to be overexpressed, we found reduced levels of *hsa-miR-185-5p* in PB of AD patients compared to the healthy control group. These findings may imply that the biological role of this miRNA in PB differs from that in TC. More study is needed to validate these findings. In a newly published study (Lugli et al., 2015), lower amounts of *hsa-miR-185-5p* were seen in the plasma of individuals with AD when compared with healthy controls. Our findings are consistent with these results.

Expression levels of *SHISA7* and *BCAS4* were shown to have a strong positive correlation, suggesting an interacting network, possibly owing to the regulation of ceRNA in PB samples. Whereas *SHISA7* and *BCAS4* are closely correlated in a direct manner, implying an interactional network, their activities in the development of AD should be reassessed considering whole blood specimens because the altered patterns of *BCAS4* expression were not strong enough to be meaningful. Nevertheless, the results obtained are just preliminary. One possible explanation for this could be our small sample size. Contrary to the common belief that miRNAs are repressive, we discovered a weak positive correlation between *SHISA7* and *hsa-miR-185-5p* in AD patients’ PB samples. Although it is unusual, both negative and positive miRNA-mRNA correlations have been witnessed in many studies, suggesting the presence of a complicated network encompassing miRNA target inhibition (resulting in negative miRNA-mRNA correlations) besides feed-forward regulation provoked by widely known transcription factors (resulting in positive miRNA-mRNA correlations) (Friard et al., 2010; Chen et al., 2011; Chien et al., 2014; Diaz et al., 2015). The *hsa-miR-185-5p*, as previously mentioned, suppresses the expression of *SHISA7* in neuroblastoma cells (human and murine) (Marques et al., 2012). This finding is inconsistent with our result. In line with our result, another study found that *hsa-miR-3681-5p* acts as a super-enhancer by employing alternative enhancers and promoters, transcription factors, activators, mediators, and RNA Pol II. Further, its enhancing activity acts as an inhibitor of variable number tandem repeats (VNTRs) functions in the *SHISA7* 3′ UTR (Lee et al., 2020). More studies are needed to establish whether *hsa-miR-185-5p* functions as an enhancer in AD patients’ PB.

Eventually, we determined that *SHISA7* and *hsa-miR-185-5p* had a diagnostic value of 0.758 and 0.779, in turn, in differentiating patients with AD from healthy subjects. Because of the small sample size, the obtained findings should be interpreted with caution. If future research supports the findings of the present study, the *SHISA7* and *hsa-miR-185-5p* transcription levels might be utilized as AD-associated markers.

### Limitations

Our research has several limitations. Firstly, various aspects, such as diverse methods, sample preparation, platforms, data analysis, and patient characteristics, may impact expression patterns of genes. Second, a limited sample size might lead to a lack of statistical validity. Furthermore, our bioinformatics analysis must be verified by confirmatory experimental techniques. Finally, we did not examine expressions of *SHISA7*, *BCAS4*, and *hsa-miR-185-5p* in the PB cell subpopulation.

## Conclusion

Competing endogenous RNA regulation has biologically significant consequences in a variety of illnesses, which can help to explain pathogenic processes and provide possibilities for novel treatments. As a result, our attempts to comprehend various aspects of ceRNA regulation processes in AD pathogenesis give new insights into possible molecular targets and lead to the discovery of ceRNA-based biomarkers. The present study is the first evidence to highlight the expression of the *BCAS4*/*miR-185-5p*/*SHISA7* ceRNA axis in the brain and PB of AD patients. The obtained results are preliminary, and further *in vitro* and *in vivo* research might strengthen these results. Whereas the possible roles of this ceRNA axis require more exploration, this research advances the current insights into the GABAergic system associated with GABAAR and provides a novel viewpoint on the molecular processes behind AD development.

## Data Availability Statement

The original contributions presented in the study are included in the article/Supplementary Material, further inquiries can be directed to the corresponding authors.

## Ethics Statement

The studies involving human participants were reviewed and approved by Tabriz University of Medical Sciences’ Clinical Research Ethics Committee (Ethical code: IR.TBZMED.REC.1398.1264). The patients/participants provided their written informed consent to participate in this study.

## Author Contributions

HS, MR, and MoT wrote the draft and revised it. SA-J and JG analyzed the data. MA, AJ, and MaT performed the experiments. BH and RJ collected the data and corresponding clinical information. All authors read and approved the submitted version.

## Conflict of Interest

The authors declare that the research was conducted in the absence of any commercial or financial relationships that could be construed as a potential conflict of interest.

## Publisher’s Note

All claims expressed in this article are solely those of the authors and do not necessarily represent those of their affiliated organizations, or those of the publisher, the editors and the reviewers. Any product that may be evaluated in this article, or claim that may be made by its manufacturer, is not guaranteed or endorsed by the publisher.

## Footnotes

1
https://www.ncbi.nlm.nih.gov/geo/

2
https://www.bioconductor.org/
