# Supplementary material for: Identification and Analysis of BCAS4/hsa-miR-185-5p/SHISA7 Competing Endogenous RNA Axis in Late-Onset Alzheimer’s Disease Using Bioinformatic and Experimental Approaches
Source: Front Aging Neurosci. 2022 Feb 21;14:812169. doi: 10.3389/fnagi.2022.812169 (PMC8899724; doi:10.3389/fnagi.2022.812169)
Supplement: Supplementary file 1 [file Data_Sheet_1.docx]

**
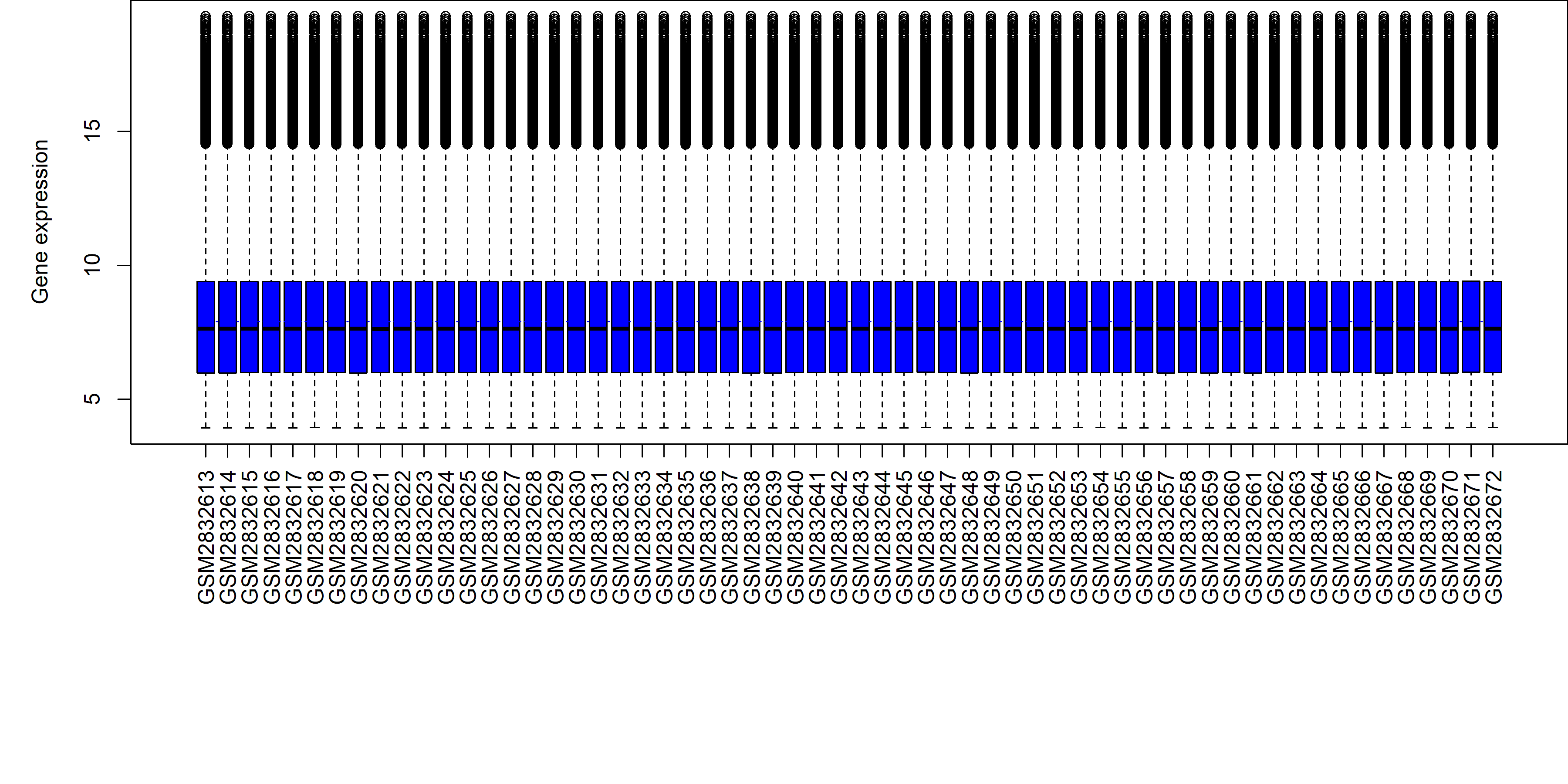
**

**Figure S1.** Boxplot for GSE106241 dataset. The horizontal axis represents the names of samples, and the vertical axis represents the gene expression.

**
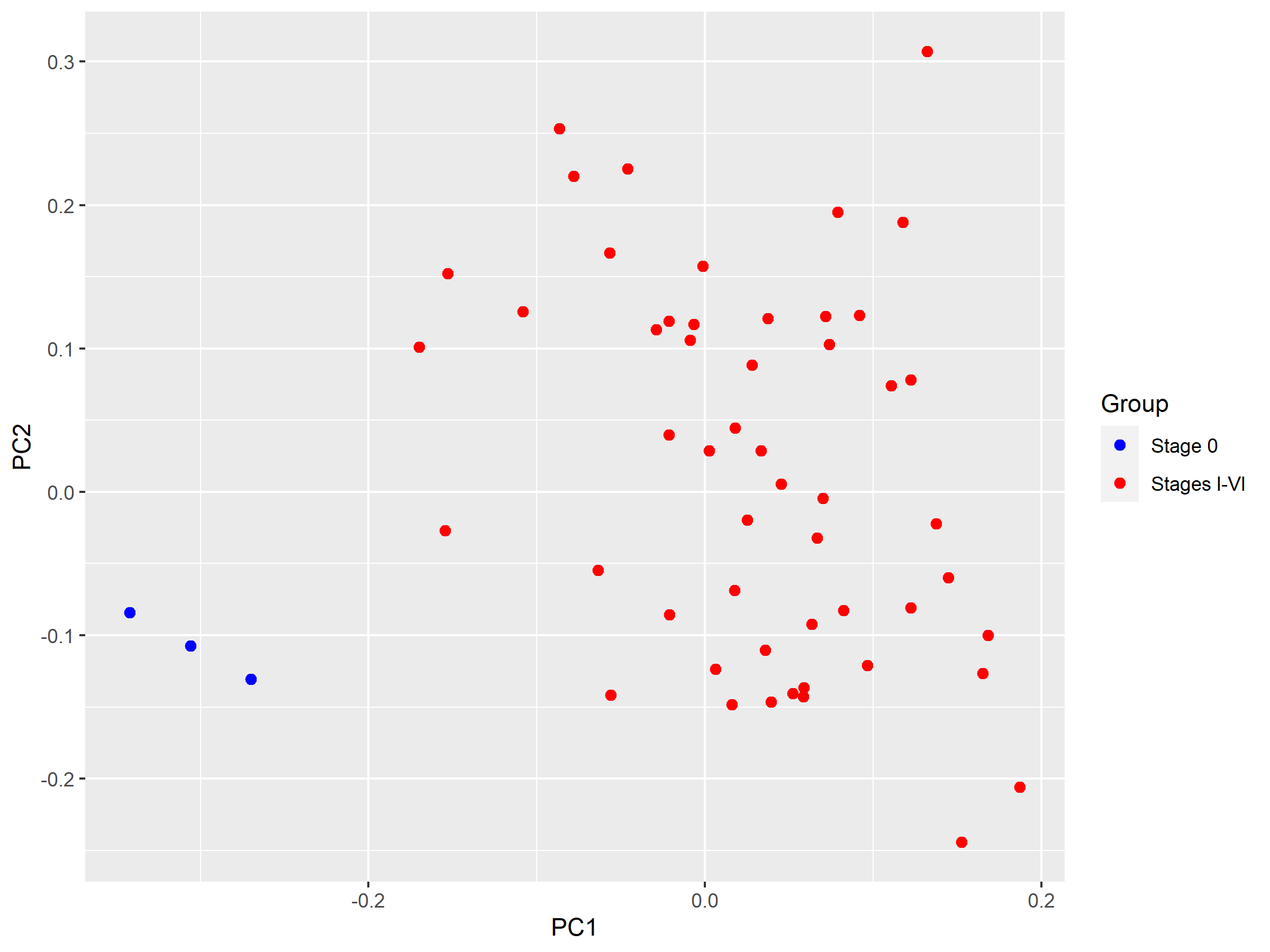
**

**Figure S2.** Principal component analysis (PCA) for the GSE106241 dataset. All samples are segregated by presence (stages I-VI) or absence (stage 0) of neurofibrillary tangles (on PC1).


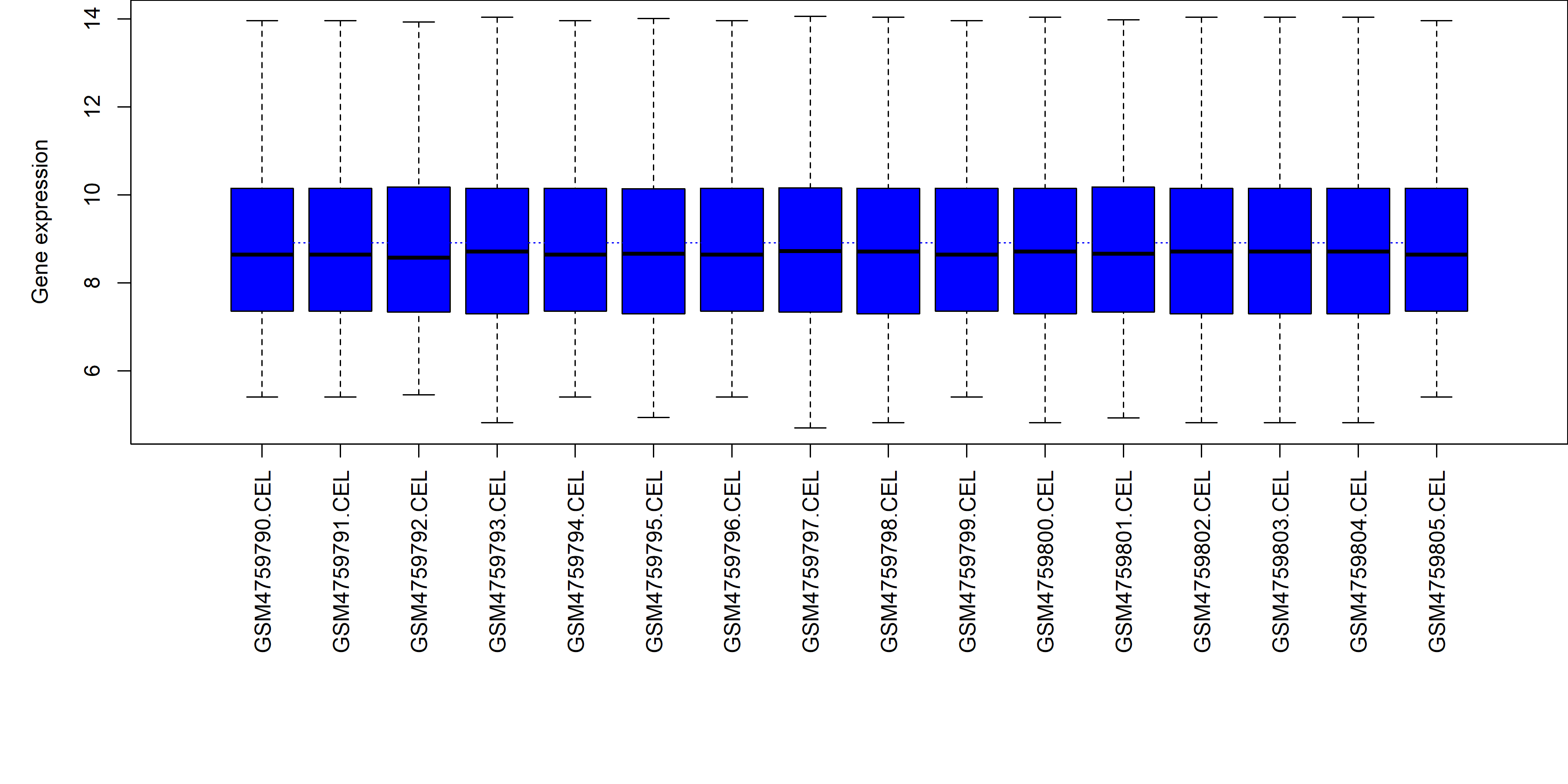


**Figure S3.** Boxplot for GSE157239 dataset. The horizontal axis represents the names of samples, and the vertical axis represents the gene expression.

**
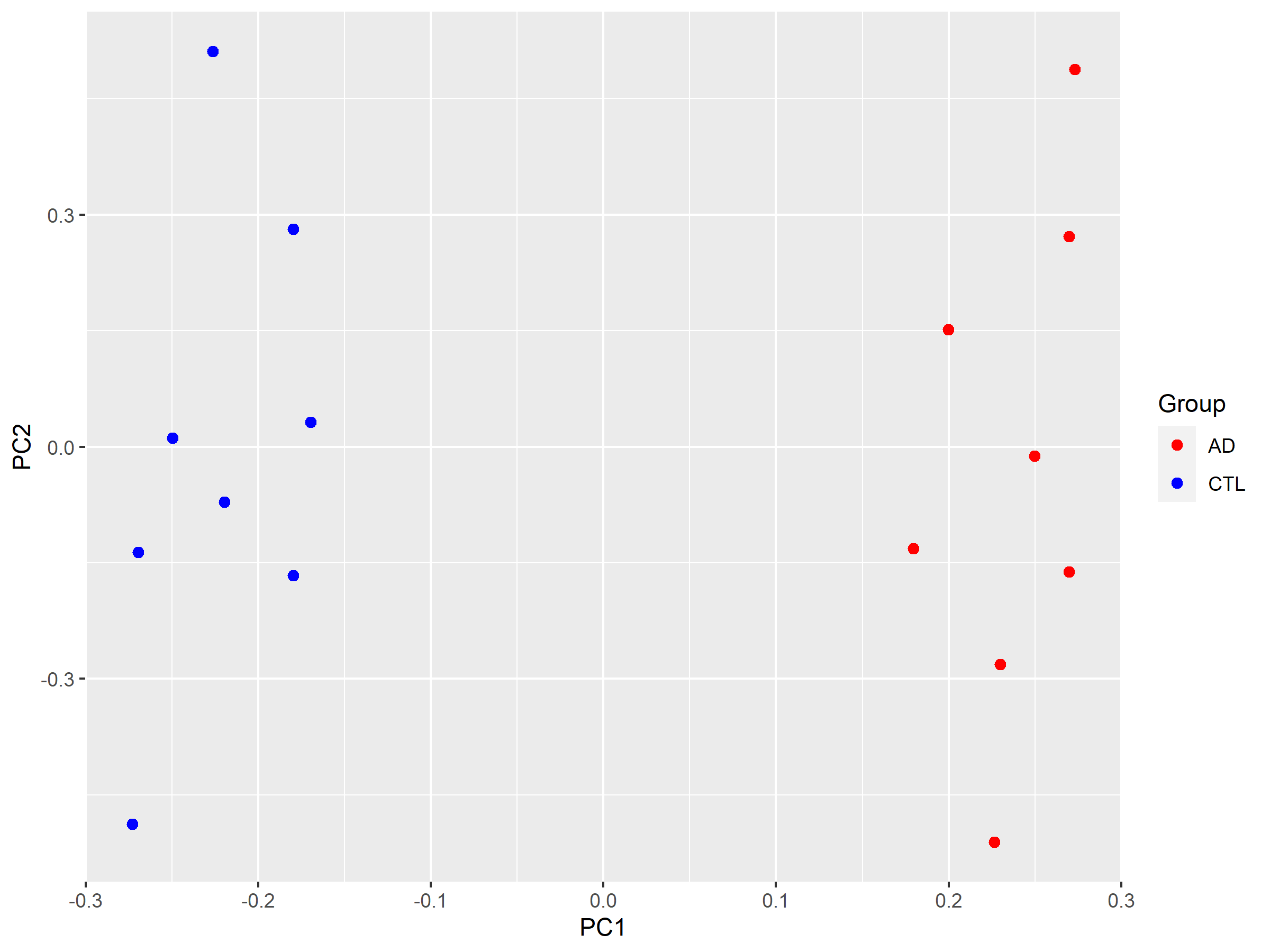
**

**Figure S4.** Principal component analysis (PCA) for the GSE157239 dataset. All samples are segregated by condition group (on PC1).
